# PKC putative phosphorylation site Ser^235^ is required for MIP/AQP0 translocation to the plasma membrane

**Published:** 2008-05-29

**Authors:** Nady Golestaneh, Jianguo Fan, Peggy Zelenka, Ana B. Chepelinsky

**Affiliations:** Laboratory of Molecular and Developmental Biology, National Eye Institute, NIH, Bethesda, MD

## Abstract

**Purpose:**

To investigate the functional significance of MIP/AQP0 phosphorylation at serine^235^.

**Methods:**

MIP/AQP0 expression and cellular localization was studied in rat lens epithelia explants induced to differentiate by FGF-2. MIP wild type (WT) and MIP (S235A) mutant expression plasmids were constructed and transiently expressed in RK13 cells. Subcellular localization of endogenous MIP in differentiating lens epithelia explants or of transfected MIP expression vectors in RK13 cells was analyzed by immunofluorescence confocal microscopy.

**Results:**

MIP/AQP0 expressed in lens epithelia explants induced to differentiate by FGF-2 localizes to the plasma membrane of elongating cells. However, MIP/AQP0 translocation to the plasma membrane was prevented by inhibiting PKC activity with Go6976, resulting in retention in the cytoplasmic compartment. This effect was specific to MIP/AQP0; localization of AQP1 to the cell membrane was not affected by Go6976. When the consensus PKC phosphorylation site at MIP Ser^235^ was mutated to alanine and transiently expressed in transfected RK13 cells, the mutant MIP was retained in the cytoplasmic compartment in contrast to WT MIP that localized to the plasma membrane of the transfected RK13 cells. Colocalization studies indicated that the mutant MIP was retained in the trans-Golgi network.

**Conclusions:**

Our results indicate that serine^235^ is required for proper intracellular transport of MIP/AQP0 from the trans-Golgi network to the plasma membrane. A PKC dependent phosphorylation event involving MIP at serine^235^ is most likely involved in this process.

## Introduction

MIP/AQP0 is the major intrinsic protein of the ocular lens. It is specifically expressed in the lens fibers. Mutations in the *MIP/AQP0* gene result in genetic cataracts in mice and humans [1-11]. In the last decade, great advances have been made in understanding the function of this protein, which is also known as “the founder of the MIP or aquaporin gene family.” Initially it was considered to be a gap junction protein. Even though it was later found not to be a member of the connexin gene family, it was demonstrated that MIP interacts transiently with some connexins and appears to be involved in gap junction formation [12-14]. It functions as a water channel when tested in various functional assays [2,15-23]. However, MIP/AQP0 has also been demonstrated to have additional functions such as acting as an adhesion molecule [24,25], forming thin junctions [26-29], and playing a role in the correct formation of sutures in the ocular lens [30-33]. In this way, MIP/AQP0 contributes to the minimal intercellular space between the lens fibers and suture formation required for optimal focusing and transparency of the lens [7,8,22,30]. The MIP COOH-terminal domain interacts with other lens proteins such as gamma crystallins [34,35], filensin, CP49 [36], and connexins [13]. Posttranslational modifications of MIP such as proteolysis [26,37,38] and phosphorylation [15,36-41] may play a role in regulating the various functions that MIP is able to play in the lens for maintaining lens transparency.

However, regulation of the function of MIP/AQP0 by signaling pathways during lens differentiation is not understood as well. We have previously demonstrated that the ERK and JNK signaling pathways are involved in the regulation of expression of the *MIP* gene during the induction of lens epithelia differentiation by FGF-2 [42]. In this study, we demonstrate that although the PKC signaling pathway does not regulate MIP transcription, it does play an essential role in the trafficking of MIP/AQP0 from the Golgi apparatus to the plasma membrane to be able to perform its physiologic function in the ocular lens.

## Methods

### Chemicals and reagents

Turbo Pfu DNA polymerase was obtained from Stratagene (La Jolla, CA). The Polymerase Chain Reaction (PCR) Purification Kit and The Plasmid Midi and Maxi Kits were purchased from Qiagen (Valencia, CA). PKC inhibitor, Go6976, was obtained from Calbiochem (San Diego, CA). Dimethylsulfoxide (DMSO) and 4’,6-diamidino-2-phenylindole dihydrochloride (DAPI) were obtained from Sigma-Aldrich (St. Louis, MO). Alexa 555-streptavidin and Alexa 488-streptavidin were from Molecular Probes (Eugene, OR). All other chemicals were reagent grade and from standard commercial sources.

### Antibodies

The MIP-specific rabbit polyclonal antiserum was obtained from Alpha Diagnostic (San Antonio, TX). Antibodies to Aquaporin 1 (mouse monoclonal), trans-Golgi network marker 38K (mouse monoclonal TGN38), goat anti-rabbit biotinylated, and anti-mouse biotinylated were purchased from Abcam (Cambridge, MA).

### Plasmids

All plasmid DNAs used were propagated in *E. coli* strain, DH5α or DH10B, and were purified by ion exchange chromatography using Plasmid Midi Kits or Endonuclease-Free Maxi Kits from Qiagen (Valencia, CA).

### Plasmid constructions

The plasmid, pCMVScript, was purchased from Stratagene. The expression vector for wild type mouse MIP (pCMV-MIP) was constructed as indicated before [35]. The expression vector for Ala^235^ mutant MIP (pCMV- MIP Ala^235^) was constructed by site-directed mutagenesis of MIP in pCMV-MIP, which was accomplished by use of Quick Change II Site-Directed Mutagenesis Kit (Stratagene) according to the manufacturer’s instructions. The introduced mutation Ala^235^ was verified by DNA sequencing.

### DNA sequencing

DNA sequencing of plasmid constructions was performed using a commercial system (CEQ DTCS-Quick Start Kit; Beckman-Coulter, Hialeah, FL) and an automated DNA analysis system (CEQ 2000XL; Beckman-Coulter) according to the manufacturer’s instructions.

### Lens epithelia explant culture

Eyes were dissected from three-day-old Sprague Dawley rats (Charles River Laboratories, Wilmington, MA). The lens capsule with adhering epithelial cells was micro-dissected from the lens fibers and pinned down around its periphery with forceps onto the surface of either a 35 mm or 60 mm culture dish in Medium 199. The resulting lens epithelia explants were pre-incubated for 1 h in Medium 199 containing either 0.2% DMSO (control) or PKC inhibitor (4 μM Go6976%–0.2% DMSO) before adding the culture medium. The lens epithelia explants (three to five explants per culture dish) were cultured according to the method previously described [42] in Medium 199 containing 0.1% BSA, 1% gentamicin, and 100 ng/ml FGF-2 (Sigma Chemical Co, St. Louis, MO) to induce differentiation [42,43] The control contained 0.2% DMSO and experimental with PKC inhibitor 4 μM Go6976 - 0.2% DMSO. The medium was changed every 24 h. All animals were treated in compliance with the guidelines of the Association for Assessment and Accreditation of Laboratory Animal Care International (AAALAC International). The study protocol was approved by the National Institutes of Health Animal Care and Use Committee.

### Cell line

The RK13 (rabbit kidney) cell line was obtained from the American Type Culture Collection and was maintained as monolayer cultures at 37 °C in a 5% CO_2_/95% air incubator in Dulbecco's Modified Eagle's Medium (DMEM) supplemented with 10% heat inactivated fetal bovine serum (FBS).

### Transfections of the RK13 cell line

After 24 h in culture, the cells were transfected with the plasmid constructions, pCMV-WT MIP and pCMV- Ala^235^ MIP (5 μg), using the Superfect Transfection Reagent (Qiagen) as previously described [35]. Cells were incubated with the transfection mixture for 5 h. The medium was changed every 24 h. Forty eight hours after transfection (three days in culture), the cell were fixed in 4% paraformaldehyde for immunocytochemistry.

### RNA isolation and reverse transcription polymerase chain reaction

Explants were removed with forceps under the dissecting microscope at the end of the culture period, and RNA was isolated from 10 explants by using the Absolutely RNA Nanoprep Kit (Stratagene, Cedar Creek, TX) according to the manufacturer’s protocol. cDNA was prepared with the SuperScript First-Strand Synthesis System for reverse transcription polymerase chain reaction (RT–PCR; Invitrogen, Carlsbad, CA).

### Relative quantification of gene expression using real-time polymerase chain reaction

Relative amounts of *MIP* mRNA and that of the control gene were quantified by real-time PCR using the ABI Prism 7900 Sequence Detection System (Applied Biosystems, Foster City, CA). Primers and probe design for the real-time PCR was made with Primer Express version 2 from Applied Biosystems. The *MIP*-specific primer pair were 5′ CAC CAG CTG TCC GAG GAA A 3′ (forward) and 5′ GCG TCA GGA AGA TCT CCA CAG T 3′ (reverse), and the probe (FAM-MGB) specific for *MIP* was FAM −5′ TCA ACA CGC TGC ATG C 3′-MGB. Primers for mouse 18S rRNA (as control) were 5′ AGT CCC TGC CCT TTG TAC ACA 3′ (forward) and 5′ CCG AGG GCC TCA CTA AAC C-3′ (reverse), and the VIC-Tamra probe sequence for 18S rRNA was 5′ CCC ATC AAC AGA GAG CGA ACT 3′. The *MIP* forward primer spans the exon 1–exon 2 boundary to avoid amplification of possible contamination of the *MIP* genomic sequence. All runs were performed in triplicate according to the default PCR protocol (50 °C for 2 min, 95 °C for 10 min, and 40 cycles of 95 °C for 15 s and 60 °C for 1 min) or the default one-step RT–PCR protocol (42 °C for 30 min, 95 °C for 10 min, and 40 cycles of 95 °C for 15 s and 60 °C for 1 min). Relative standard curves were generated for each primer set so that the input amount from unknown samples could be calculated. Expression of *MIP* was normalized with 18S rRNA expression.

### Confocal immunofluorescence

The lens explants and the transfected RK13 cell line were fixed in 4% formaldehyde in isotonic PBS (pH 7.4) buffer for 2 h at room temperature. Cellular localization of MIP, aquaporin 1, or TGN 38K either in the transfected cells or in the explants was examined by indirect immunofluorescence labeling. Briefly, the explants or the cells were incubated in ICC buffer (PBS containing 0.20% Tween 20, 0.05% sodium azide, and 0.5% BSA, pH 7.3) for 20 min at room temperature. The rat lens explants were then incubated in ICC buffer with rabbit polyclonal anti-MIP (1:50) and mouse monoclonal anti-aquaporin 1 (1:50) for 1 h at 37 °C. The RK13 cells were incubated with rabbit polyclonal anti-MIP (1:50) and mouse monoclonal anti-TGN 38K (1:50) for 1 h at 37 °C. The explants or the cells were washed in ICC buffer followed by incubation for 1 h in ICC buffer containing fluorescent dye-conjugated secondary antibodies (goat anti-rabbit biotinylated and Alexa 555-streptavidin or anti-mouse biotinylated and Alexa 488-streptavidin) along with either DAPI or SYTOX green dye. Extensive washing was performed after each incubation step. After washing, the samples were mounted with TBS/glycerol (1:1) containing *p*-phenylenediamine (Sigma-Aldrich, St. Louis, MO) and covered by a coverslip. Samples were stored at 4 °C until they were imaged by confocal fluorescence microscopy.

The stained and mounted cells in 60 mm culture dishes were imaged using a Leica TCS SP2 confocal microscope (Leica Microsystems, Exton, PA) with a Leica 40X HCX Plan Apo CS 0.85 NA and a Leica 40X Oil HCX Plan Apo CS 0.75–1.25 NA objective lens.

## Results

### PKC inhibitor does not affect *MIP* transcription in rat lens explants

We have previously shown that induction of rat lens epithelia explant differentiation into lens fibers by FGF-2 involves the activation of transcription of the *MIP/AQP0* gene through the activation of the FGF downstream signaling components, ERK and JNK [42].

**Figure 1:**
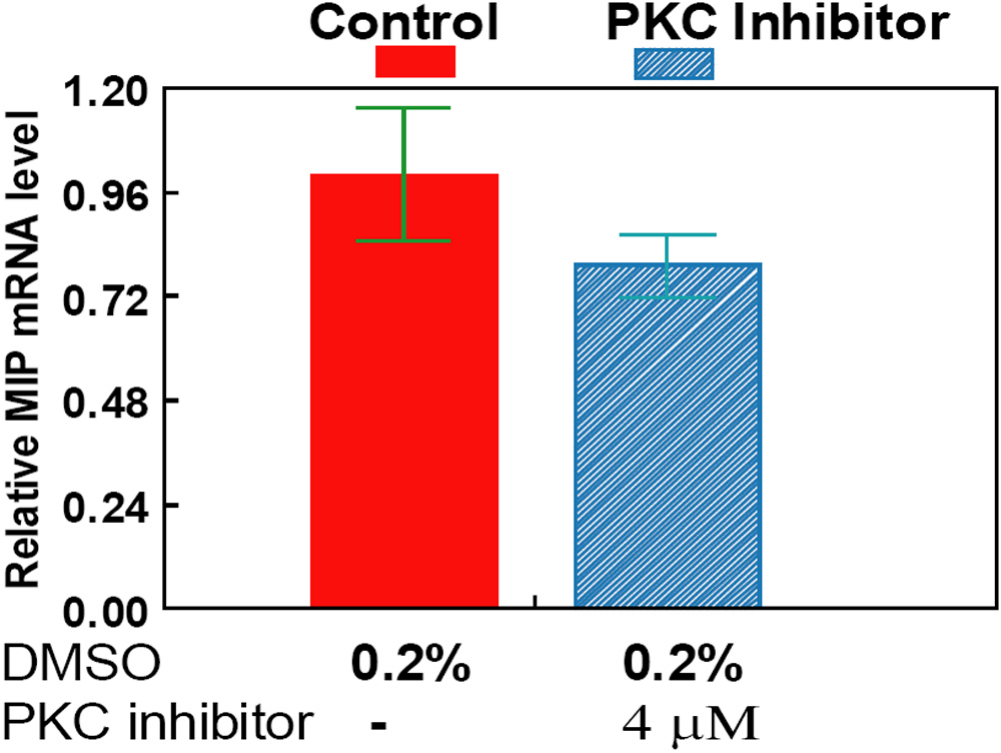
PKC inhibitor does not affect *MIP* transcription in rat lens explants. Real-time PCR analysis is shown of *Mip* mRNA from rat lens epithelia explants cultured for 72 h with 100 ng/ml FGF-2 in the absence (red) or presence of Go6976 (4 μM; blue hatched) as indicated in Methods. Standard deviations are indicated in green. The endogenous *Mip* mRNA level is not significantly affected in rat lens explants treated with PKC inhibitor, Go6976.

To examine whether PKC signaling was also involved in activating *MIP* transcription during lens epithelia differentiation induced by FGF-2, we investigated whether the synthetic inhibitor to PKC, Go 6976 [44], would affect *MIP* mRNA levels. We quantitated the *MIP* mRNA amounts in the untreated control and PKC inhibitor-treated explants using real-time PCR analyses. As shown in Figure 1, no significant change in *MIP* transcript levels was observed in the control and PKC inhibitor-treated explants. These results imply that unlike ERK and JNK, PKC signaling (at least through isoenzymes α, γ, and μ [44,45]) does not contribute to *MIP* transcription.

### PKC inhibitor affects MIP plasma membrane localization in rat lens explants

**Figure 2:**
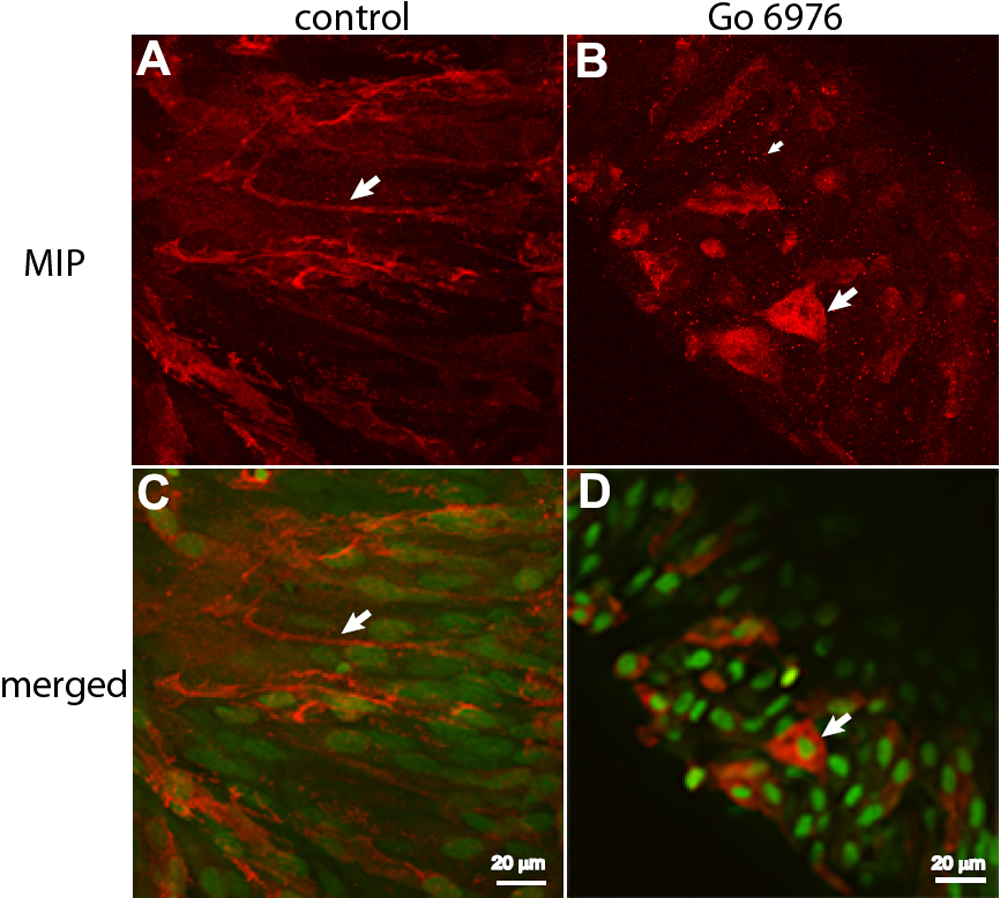
PKC inhibitor prevents MIP membrane localization in differentiating lens explants. MIP immunofluorescence (red) shows MIP localization in the cell membrane (**A,C**; indicated with large arrows) of rat lens epithelial explants cultured in 100 ng/ml FGF-2 and 0.2% DMSO for 72 h (control; as described in Methods; **A** and **C**). MIP is retained in the cytosolic compartment (**B,D**; large arrows point to cell with strong MIP signal in the cytoplasmic compartment; numerous MIP-positive vesicles were also seen, small arrow) in rat lens epithelial explants cultured for 72 h (100 ng/ml FGF-2 in the presence of 4 μM Go6976 plus 0.2% DMSO as described in Methods; panels **B** and **D**). **C** and **D** show the merged images of MIP immunofluorescence (**A** and **B**, respectively) with the ones for green nuclei staining. Green fluorescence indicates nuclei stained with SYTOX green dye. Scale bars represent 20 μm. **C** and **D** show one of the z stack confocal images of MIP immunofluorescence cell distribution through the thickness of the cultured explant in the control (animation 1) and Go6976 (animation 2) experiments, respectively.

Our results show that PKC is not involved in regulating MIP at the level of transcription. As PKC has been reported to have a possible role in MIP phosphorylation [46], we set up to investigate the role of PKC as a potential contributor to the subcellular localization of MIP. Thus, we studied the effect of the PKC inhibitor, Go6976, in the localization of expressed MIP in rat lens epithelia explant cultures induced to differentiate by FGF-2. As we previously demonstrated [42], MIP expressed in rat epithelia explants and induced to differentiate by FGF-2 localizes to the plasma membrane of elongating cells that characterize the first stages of lens epithelia differentiation as shown in Figure 2A,C and animation 1. However, when the PKC inhibitor, Go6976, is present during FGF-2-induced lens epithelia explants differentiation, MIP immunofluorescence shows that cells in explants treated with the PKC inhibitor retained MIP in the cytosolic compartment (Figure 2B,D and animation 2). These results implied the importance of PKC isoenzymes (α, γ, and/or μ [44,45]) in MIP targeting to the plasma membrane in differentiating lens cells.

### Inhibition of PKC perturbs MIP/AQP0 translocation to the plasma membrane but does not affect AQP1 localization to the plasma membrane of rat lens explants

**Figure 3:**
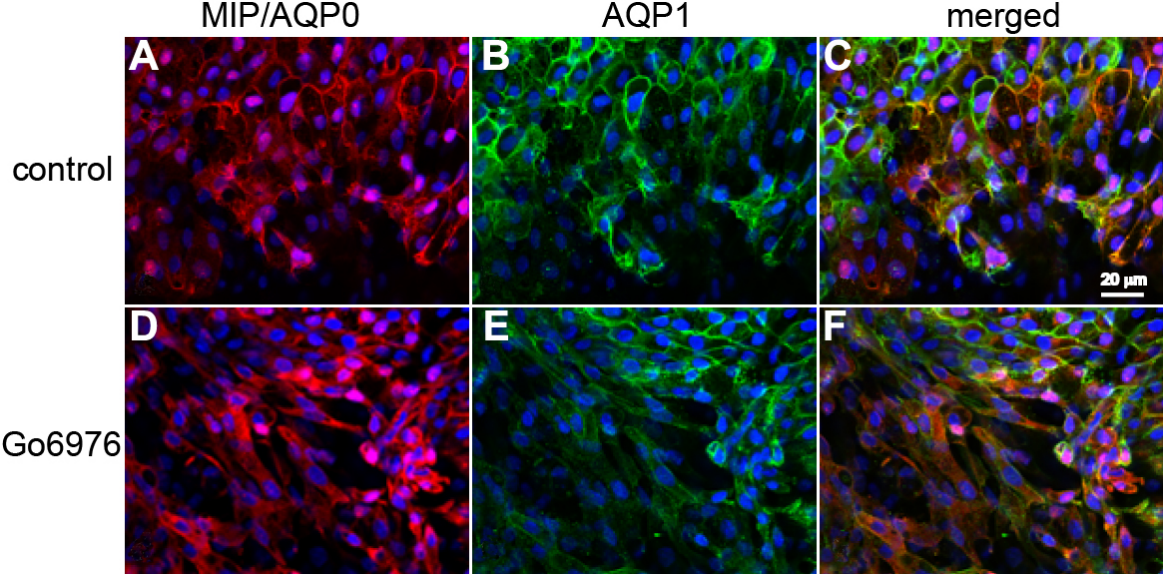
PKC inhibitor perturbs MIP/AQP0 membrane localization without affecting AQP1 membrane localization in differentiating lens explants. Images are shown of MIP/AQP0 immunofluorescence (red; **A**) and AQP1 immunofluorescence (green; **B**) in rat lens epithelial explants cultured in 100 ng/ml FGF-2 and 0.2% DMSO for 72 h (control, as described in Methods). Images are shown of MIP/AQP0 immunofluorescence (red; **D**) and AQP1 immunofluorescence (green; **E**) in rat lens epithelial explants cultured in 100 ng/ml FGF-2 in the presence of 4 μM Go6976 plus 0.2% DMSO for 72 h (as described in Methods). **C** and **F** show the merged images of MIP/AQP0 immunofluorescence and AQP1 immunofluorescence and the DAPI nuclear staining in controls (**A** and **B**) and Go6976 experiments (**D** and **E**), respectively. Scale bars represent 20 μm.

To distinguish whether the inhibition of PKC isoenzymes by Go6976 [44,45] specifically affects the localization of MIP to the plasma membrane or if it is the result of a general effect on membrane proteins due to other pathways being affected in the differentiating lens epithelia cells, we also looked at the possible effect of the PKC inhibitor Go6976 on the localization of AQP1. AQP1 is expressed in the anterior and equatorial lens epithelia. As the lens epithelial cells start differentiating into lens fibers, AQP1 expression is turned off and MIP/AQP0 expression is turned on [22]. The induction of lens epithelia differentiation by FGF-2 into lens fibers in the rat explant system allows us to study the expression of both proteins during this transition period. As shown in Figure 3A,B, both MIP/AQP0 and AQP1 are expressed and localized to the plasma membrane in the lens explant cells. When the explants are incubated in the presence of Go6976, MIP/AQP0 accumulates in the cytoplasmic compartment (Figure 3D) whereas AQP1 localization into the cell plasma membrane is not affected (Figure 3E). These results clearly demonstrate that PKC isoenzymes inhibited by Go6976 (α, γ, and/or μ [44,45]) affect only MIP/AQP0.

### Mutation of PKC putative phosphorylation site (Ser^235^) prevents MIP targeting to the cell plasma membrane

**Figure 4:**
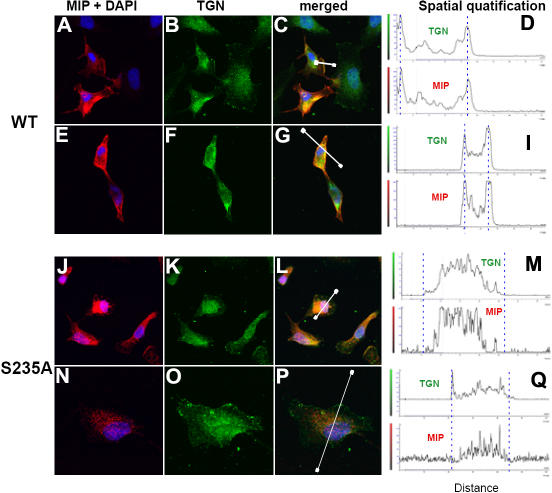
Mutation of PKC putative phosphorylation site (Ser^235^) prevents MIP translocation from the trans-Golgi network to the plasma membrane. Immunofluorescence of RK13 cells transfected with pCMV- MIP (WT; **A**-**I**) or pCMV-MIP Ala^235^ (S235A; **J**-**Q**) for 72 h is shown. **A** and **E** as well as **J** and **N** show the merged images of MIP red immunofluorescence with the corresponding images of their DAPI nuclear staining. **B** and **F** as well as **K** and **O** show the trans-Golgi network marker 38K (TGN) green immunofluorescence. **C, G, L,** and **P** show the merged images of MIP immunofluorescence and DAPI nuclear staining with their respective TGN green immunofluorescence (**A** and **B**; **E** and **F**; **J** and **K**; **N** and **O**, respectively). Spatial quantification was performed along a path across the plasma membrane, indicated by a white line with prominent end points in the merged images (**C**, **G**, **L**, and **P**). Red and green fluorescence was quantified separately and plotted as a function of distance along the path (**D**, **I**, **M**, and **Q**). Blue broken lines in the spatial quantification graphs indicate the approximate location of the plasma membrane (except the left line in **D**, which corresponds to the TGN region in **C**). Note that WT MIP and TGN vesicles colocalize at the plasma membrane (peaks are indicated with blue lines in **I** and right peak in **D**). MIP Ala^235^ mutant (S235A) does not colocalize with TGN vesicles at the plasma membrane (blue lines; **M** and **Q**). **C** and **G** show colocalization (yellow) of WT MIP (red immunofluorescence) and TGN 38K (green immunofluorescence) in the cytoplasmic compartment in addition to the localization of WT MIP in the plasma membrane. **J** and **N** show MIP Ala^235^ mutant punctate distribution in the cytosolic compartment (red immunofluorescence) and colocalization (yellow) with trans-Golgi network 38K (green) in **L** and **P**. **A** and **E** as well as **J** and **N** show cell images from either duplicate experiments or in different fields of the same cell culture of WT or MIP Ala^235^ mutant (S235A), respectively. Scale bars represent 10 μm. Note that three cells in **A**, **B**, and **C** (one cell at the right side and two cells in the upper part of the panels) that did not uptake the transfected MIP expression plasmid served as negative controls. They show no red immunofluorescence in contrast to two transfected cells showing the red immunofluorescence. All the cells in the panel (transfected and non-transfected) show green immunofluorescence for TGN38.

It has been reported that PKC is involved in the phosphorylation of MIP [46]. We identified MIP Ser^235^ as a potential PKC phosphorylation site in the MIP protein by analyzing it with the Scansite program [47]. Serine^235^ coincides with the major phosphorylated site identified in MIP in vivo in the lens of several species [38,39,41]. Based on this identification, the putative PKC phosphorylation site, Ser^235^, was mutated to Ala^235^ in the *MIP* cDNA. As we have previously demonstrated that MIP transiently expressed in RK13 cells localizes to the plasma membrane [35], we investigated whether the Ala^235^ mutation of MIP Ser^235^ would affect the localization to the plasma membrane when expressed in these cells. Therefore, RK13 cells were transfected with either the WT or mutant *MIP* cDNA expression vectors. Immunofluorescence of the transfected cells was performed to identify the localization pattern of the expressed MIP. In cells transfected with WT *MIP* cDNA, the translated MIP protein was localized to the cell plasma membrane (Figure 4A,E). However, in cells expressing the MIP-Ala^235^, a cytoplasmic retention of the protein was evident; the bulk of the mutant protein shows a punctate expression in the intracellular compartment and no localization in the plasma membrane (see Figure 4J,N). These results show that mutation of a single PKC phosphorylation site in the MIP protein prevents its targeting to the plasma membrane.

Membrane proteins synthesized in the endoplasmic reticulum undergo proper folding and oligomerization before being routed through the Golgi apparatus and the Golgi vesicles to their final destination in the plasma membrane. Therefore, we investigated whether the WT and mutant MIP expressed in the transfected RK 13 cells were differentially processed in subcellular compartments such as trans-Golgi vesicles. We used an antibody to the trans-Golgi network (TGN) marker 38K along with the MIP antibody to determine whether they colocalized. The 38K TGN marker, an integral protein predominantly localized to the TGN, is also a component of the TGN-derived vesicles in route to the plasma membrane and can be observed at the plasma membrane. WT MIP colocalizes with the TGN vesicles in the plasma membrane as shown in Figure 4I (peaks indicated with blue lines) and Figure 4D (right peak indicated with blue line); it also shows colocalization with the trans-Golgi network in the cytoplasmic compartment in route to the plasma membrane (Figure 4; yellow pattern in Figure 4C and Figure 4G; left peak indicated with blue line in Figure 4D). In contrast, in cells expressing the MIP mutant Ala**^235^**, MIP is absent from the plasma membrane, showing a punctate distribution (Figure 4J,N). There is colocalization with the trans-Golgi network only in the cytoplasmic compartment (Figure 4; yellow pattern in Figure 4L and Figure 4P) besides localization in other cytoplasmic vesicles. There is no colocalization of MIP mutant Ala**^235^** with the TGN vesicles at the cell plasma membrane (Figure 4M,Q). These results demonstrate that the PKC putative phosphorylation site Ser**^235^** plays a prominent role in the process of MIP translocation from the TGN to the plasma membrane by the TGN vesicles.

## Discussion

The PKC pathway is one of the major signal transduction pathways that regulate a multitude of biologic functions. Most, if not all, membrane proteins continuously shuttle between several organelles along microtubules and actin cytoskeleton [48,49]. The dynamic equilibrium of this protein subcellular transport is regulated by several factors that include the activity of protein kinases and phosphatases; PKC is one of the protein kinases implicated in this trafficking [50-53]. Several members of the PKC family are expressed in the lens [54-56]. The role of the PKC γ isozyme on the lens gap junction function has been well documented [54-63], and PKC α isozyme may regulate the interaction of tropomodulin with cytoskeletal components in the lens [64].

PKC may be a possible contributor to MIP phosphorylation [46], and phosphorylated MIP is differentially distributed in the lens cortex and nucleus of the human lens [37]. In this study, we examined the role of active PKC on MIP expression and subcellular localization in differentiating lens cells. Our real-time PCR results which showed that the level of MIP transcripts in rat lens explants were unaffected by a PKC inhibitor to isozymes α, γ, and μ indicated that these PKC isozymes are not required for MIP gene expression. However, we found that active PKC isozymes (α, γ, and/or μ) play an important role in MIP translocation to the plasma membrane. PKC inhibition prevents MIP integration in the plasma membrane, resulting in retention in the cytoplasmic compartment of lens epithelia explants that were induced to differentiate by FGF-2. This effect was specific for MIP/AQP0; AQP1 membrane localization in the lens explants was not affected by the inhibitor to PKC isoenzymes α, γ, and μ.

We then focused on MIP serine^235^, a putative PKC phosphorylation site that has also been identified as a major MIP phosphorylation site in rat, bovine, and human lens [38,39,41]. Our results showed that mutation of MIP serine^235^ to alanine^235^ prevents translocation of MIP-Ala^235^ mutant from the trans-Golgi network to the plasma membrane of transfected RK13 cells. The additional punctate distribution of the mutant MIP in the cytoplasmic compartment probably represents its targeting from the trans-Golgi to the degradation pathways (i.e., lysosomal vesicles).

Natural mutations in *MIP*, either deletions or substitutions, have been linked to genetic cataracts with a dominant phenotype in mice and humans [1-11]. Some of those MIP mutants with mutations involving transmembrane domains are retained in the endoplasmic reticulum [5,6,8,9]. As MIP assembles as a tetramer [26,28], these MIP mutations prevent oligomerization and correct folding required for MIP transport from the endoplasmic reticulum to the Golgi apparatus. Our present results, which indicate that phosphorylation of MIP at serine^235^ is required for proper MIP translocation from the trans-Golgi network to the plasma membrane, reveal a novel control point in the MIP trafficking possibly serving as a sorting signal. PKC-dependent serine phosphorylation has also been identified as Golgi sorting signals of other proteins such as NMDA receptors and phosphatidylinositol transfer protein β [65-68]. As PKC μ/PKD localizes to the trans-Golgi network and regulates vesicle formation required for trafficking of membrane proteins to the plasma membrane [69,70], it is tempting to speculate that PKC μ may play a role in MIP trafficking to the plasma membrane from the trans-Golgi network.

MIP/AQP0 serine^235^ is located in the COOH-terminal domain, which is involved in interactions with other proteins in the lens such as gamma crystallins [34,35], filensin, CP49 [36], connexins [13], and calmodulin [71]. Phosphorylation of MIP COOH-terminal domain reduces its affinity for calmodulin [71], known to regulate water channel activity in functional assays [20,21,71]. Thus, phosphorylation of MIP/AQP0 at Ser^235^ may be involved in regulating its functions once integrated in the plasma membrane. We have now demonstrated in this study that MIP phosphorylation at Ser^235^ is required for correct trafficking to the plasma membrane; therefore, this phosphorylation event plays a primary role in MIP function by being required for proper targeting to the plasma membrane. Once MIP/AQP0 is correctly integrated in the plasma membrane, it becomes enabled to accomplish its functions in the lens, such as water channel, adhesion molecule and/or other possible functions required for the optical properties of the normal lens.

## Supplementary Material

Animation 1

Animation 2
